# Bedside Lung Ultrasound During Acute Chest Syndrome in Sickle Cell Disease

**DOI:** 10.1097/MD.0000000000002553

**Published:** 2016-02-18

**Authors:** Keyvan Razazi, Jean-François Deux, Nicolas de Prost, Florence Boissier, Elise Cuquemelle, Frédéric Galactéros, Alain Rahmouni, Bernard Maître, Christian Brun-Buisson, Armand Mekontso Dessap

**Affiliations:** From the Assistance Publique-Hôpitaux de Paris, Hôpitaux Universitaires Henri Mondor, DHU A-TVB, Service de Réanimation Médicale (KR, NdP, FB, EC, CB-B, AMD); UPEC, Faculté de Médecine de Créteil, IMRB, GRC CARMAS (KR, NdP, BM, CB-B, AMD); UPEC, Faculté de Médecine de Créteil (J-FD, FG, AR); Assistance Publique-Hôpitaux de Paris, Hôpitaux Universitaires Henri Mondor, Service de Radiologie (J-FD, AR); Assistance Publique-Hôpitaux de Paris, Hôpitaux Universitaires Henri Mondor, Unité des Maladies Génétiques du Globule Rouge (FG); and Assistance Publique-Hôpitaux de Paris, Hôpitaux Universitaires Henri Mondor, DHU A-TVB, Antenne de Pneumologie, Service de Réanimation Médicale (BM), Créteil, France.

## Abstract

Lung ultrasound (LU) is increasingly used to assess pleural and lung disease in intensive care unit (ICU) and emergency unit at the bedside. We assessed the performance of bedside chest radiograph (CR) and LU during severe acute chest syndrome (ACS), using computed tomography (CT) as the reference standard.

We prospectively explored 44 ACS episodes (in 41 patients) admitted to the medical ICU. Three imaging findings were evaluated (consolidation, ground-glass opacities, and pleural effusion). A score was used to quantify and compare loss of lung aeration with each technique and assess its association with outcome.

A total number of 496, 507, and 519 lung regions could be assessed by CT scan, bedside CR, and bedside LU, respectively. Consolidations were the most common pattern and prevailed in lung bases (especially postero-inferior regions). The agreement with CT scan patterns was significantly higher for LU as compared to CR (κ coefficients of 0.45 ± 0.03 vs 0.30 ± 0.03, *P* < 0.01 for the parenchyma, and 0.73 ± 0.08 vs 0.06 ± 0.09, *P* < 0.001 for pleural effusion). The Bland and Altman analysis showed a nonfixed bias of −1.0 (*P* = 0.12) between LU score and CT score whereas CR score underestimated CT score with a fixed bias of −5.8 (*P* < 0.001). The specificity for the detection of consolidated regions or pleural effusion (using CT scan as the reference standard) was high for LU and CR, whereas the sensitivity was high for LU but low for CR. As compared to others, ACS patients with an LU score above the median value of 11 had a larger volume of transfused and exsanguinated blood, greater oxygen requirements, more need for mechanical ventilation, and a longer ICU length of stay.

LU outperformed CR for the diagnosis of consolidations and pleural effusion during ACS. Higher values of LU score identified patients at risk of worse outcome.

## INTRODUCTION

Sickle cell disease is 1 of the most common severe monogenic disorders worldwide.^1,2^ Acute chest syndrome (ACS) is a major complication of sickle cell disease and a significant cause for morbidity and mortality in adult patients.^3–5^ ACS is characterized by fever and/or respiratory symptoms with new pulmonary infiltrates. Since ACS is grossly underestimated by physical examination alone,^6^ empiric lung chest radiograph (CR) is routinely obtained in patients suspected of ACS. In a recent report, however, we found that bedside CR had a good sensitivity but a low specificity for the radiological diagnosis of ACS.^7^

Ultrasonography is increasingly used at the bedside to assess lung disease in acutely ill patients. Lung ultrasound (LU) is a noninvasive and radiation-free point-of-care tool capable of accurately depicting lung and pleural pathologic entities.^8^ The diagnostic performance and prognostic value of LU has never been tested during ACS. The primary aim of the present study was to assess whether LU could be of help for ACS imaging and test whether lung ultrasonographic features would be associated with the outcome of ACS. Bedside CR and LU were compared to computed tomography (CT) scan, taken as the reference standard.

## METHODS

### Patients

Consecutive adults (≥18 years) with sickle cell disease admitted to the medical intensive care unit (ICU) of Henri Mondor University Hospital (Créteil, France) between June 2011 and April 2014 with a diagnosis of ACS were prospectively included. The genotype of the patients had been previously determined by standard laboratory methods, including globin gene sequencing when necessary. The diagnosis of ACS by the attending physician was based on the association of a respiratory symptom (dyspnea or chest pain), abnormal lung sounds at auscultation, and a new pulmonary opacity on the CR.^7,9^ In patients diagnosed with ACS, a CT scan was ordered by the attending physician to search for pulmonary artery thrombosis,^9^ unless there was a pregnancy or contraindication to iodinated agents. The study was approved by the institutional review board of the French Society for Respiratory Medicine (Société de Pneumologie de Langue Française) as a component of standard care, and written and oral information was given to the patients. All patients received a uniform standardized treatment protocol for ACS.^10^ In patients with persistent respiratory distress despite red-blood-cell transfusion or partial-exchange transfusion, noninvasive mechanical ventilation was started; orotracheal intubation and invasive ventilation were used in patients who failed noninvasive ventilation.^11^

### Lung Imaging

Bedside LU was performed with a Philips iE33 apparatus using a 3 to 5 MHz probe (Philips ultrasound, Bothell, WA, USA). Each lung was divided into 6 regions (the upper and lower parts of the anterior, lateral, and posterior regions), as previously described.^12^ All 12 lung regions were examined via a transthoracic route with the patient in the supine or lateral position.^12^

All CT scan examinations were performed using a 64-row multidetector CT (Lightspeed VCT, General Electric , Chalfont Saint Giles UK)) , with standardized injection protocol and parameters, as previously described (600 mA, 120 kV, collimation of 0.625 mm, reconstruction slice thickness of 0.625 mm, pitch of 1, and rotation time of 500 ms, axial scan performed from the lung apices to the diaphragm, typical scan time of 4 s, approximate median CT volume dose index of 721 mGy).^7^ Each lung was divided into 6 regions (the upper and lower parts of the anterior, lateral, and posterior regions), using a cephalocaudal mid-axillary line and a transversal hilar line for delineation, and the apex, mediastinum border, diaphragm, and external limit of the rib cage as external landmarks, as previously described.^12^

Anterior bedside CRs were obtained by using a Mobilett Plus apparatus (Siemens, Saint-Denis, France), in supine or semirecumbent position, with standardized parameters (65 kV, 4–8 mA according to body mass index) and focus-film distance (1 m).^7^ The 12 lung regions that were explored by LU and CT were also analyzed using CR, using the same anatomic landmarks (apex, mediastinum border, diaphragm, external limit of the rib cage, mid-axillary line, and transversal hilar line), with posterior lung regions being defined as those with radiologic signs erasing the mediastinum border (“silhouette sign”), as previously described.^12^

### Image Analysis

Numeric bedside CR images were first interpreted by attending physicians as part of their daily clinical work (a new pulmonary opacity was required for ACS diagnosis).^9^ CR and CT images underwent a subsequent review at the end of the study by a senior radiologist expert in the field of ACS without knowledge of the initial CR interpretation and of the clinical data. Bedside LU was performed and interpreted by a trained operator (competence in critical care ultrasonography) blinded to CR and CT scan results. In addition, 115 LU recordings (from 10 patients) were randomly selected from the study and separately reviewed by 2 different trained ultrasonographers to assess inter-rater reproducibility of LU.

Three patterns of lung parenchyma (consolidation, ground-glass opacity, and normal) were defined on CT and CR for each region explored, according to Fleischner Society Glossary of terms for Thoracic Imaging^13^ and as previously described.^7^ Briefly, consolidation was defined as an homogeneous increase in pulmonary parenchymal attenuation that obscured the margins of vessels and airway walls (an air bronchogram might be present). Ground-glass opacity was defined on CR as an area of hazy increased lung opacity, usually extensive, within which margins of pulmonary vessels may be indistinct; on CT scans, it was defined as hazy increased opacity of the lung, with preservation of bronchial and vascular margins. Lung ultrasonograms were also classified in 3 categories as previously described.^12^ Consolidation corresponded to a tissular pattern frequently containing hyperechoic punctiform images representative of air bronchograms; ground-glass opacities were defined as the presence of coalescent B lines; a normal pattern was defined as the presence of noncoalescent B lines or the presence of lung sliding with A lines. A lines were defined as horizontal lines, arising from and parallel to the pleural line. B lines were defined as vertical lines, arising from and perpendicular to the pleural line; B lines were deemed coalescent if they were 3 mm apart or less. Each lung region was attributed 0, 1, or 2 points, according to the pattern of lung aeration (normal, ground-glass, or consolidation, respectively). A score was computed by summing points from the 12 regions by each lung imaging technique, to assess global lung loss of aeration, as previously suggested.^14^

Pleural effusion was defined on CR as the presence in the lower lung regions of a homogeneous opacity in which bronchovascular markings were visible with a blunting of the diaphragmatic cupula and/or a thickening of the pleural surface laterally; on CT as a homogeneous and peripheral opacification free of any air bronchograms and characterized by a CT attenuation lower than the CT attenuation of adjacent alveolar consolidation; and on LU as a dependent collection limited by the diaphragm and the pleura, with an inspiratory movement of the visceral pleura from depth to superficies.^12^ Pleural effusion was quantified on LU using the interpleural distance as previously proposed.^15^ When pleural effusion was small, we excluded isolated subpleural consolidation using M-mode as previously described.^16^

### Statistical Analysis

Data were analyzed using the IBM SPSS Statistics 19.0 statistical software package (SPSS, Armonk, NY) and R 2.15.2 (The R Foundation for Statistical Computing, Vienna, Austria). Continuous data were expressed as median (25th–75th percentiles) or mean ± standard deviation, as appropriate, and compared using the Mann–Whitney test. Categorical variables, expressed as percentages, were evaluated using the χ^2^ test or Fisher exact test. Correlations were assessed with the Spearman rho correlation coefficient. We tested the performance of reviewed bedside CRs and LUs for detecting lung parenchymal and pleural abnormalities associated with ACS, using CT scan as the reference. The agreement between imaging modalities and between blinded readers of LU recordings was estimated using the Cohen chance-corrected κ coefficient^17^ and compared using the cocor package.^18^ The agreement evaluation of scores assessing lung loss of aeration was performed according to the methods devised by Bland and Altman,^19^ with estimation of bias and limits of agreement by using CT as the reference. Two-sided *P* values <0.05 were considered significant.

## RESULTS

### ACS Episodes

Among 84 patients screened for ACS during the study period, 43 were excluded because of 1 of the following reasons: CT scan was not performed because not ordered by the attending intensivist (n = 8) or because of a contra-indication (n = 2, including 1 case of allergy and 1 case of renal failure); CT scan was performed more than 24 h before ICU admission (n = 15); LU machine and/or sonographer was unavailable within 24 h of CT scan (n = 16); and patient clinical condition worsened between CT scan and LU (eg, need for mechanical ventilation, n = 2). Thus the present study comprises 41 patients with 44 ACS episodes. Thirty-eight patients had a single ACS episode, and 3 had 2 episodes (5, 19, and 22 months apart). One patient had hematological and hemoglobin profiles indicating SC disease (double heterozygote for hemoglobin S and hemoglobin C), 1 had S-β thalassemia disease (double heterozygote for hemoglobin S and beta-thalassemia) and all others had SS disease (homozygote for the beta S globin; Table 1).

**TABLE 1:**
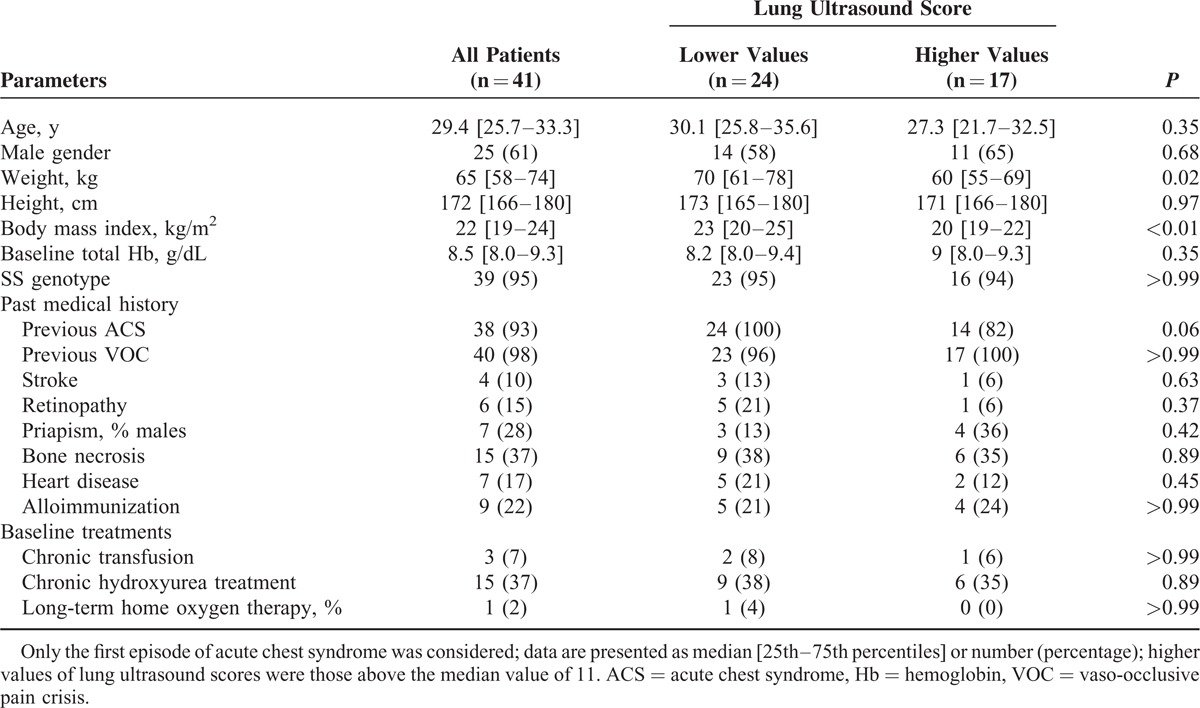
Baseline Characteristics of 41 Patients With Acute Chest Syndrome, According to the Lung Ultrasound Score

### Lung Imaging Findings

**FIGURE 1:**
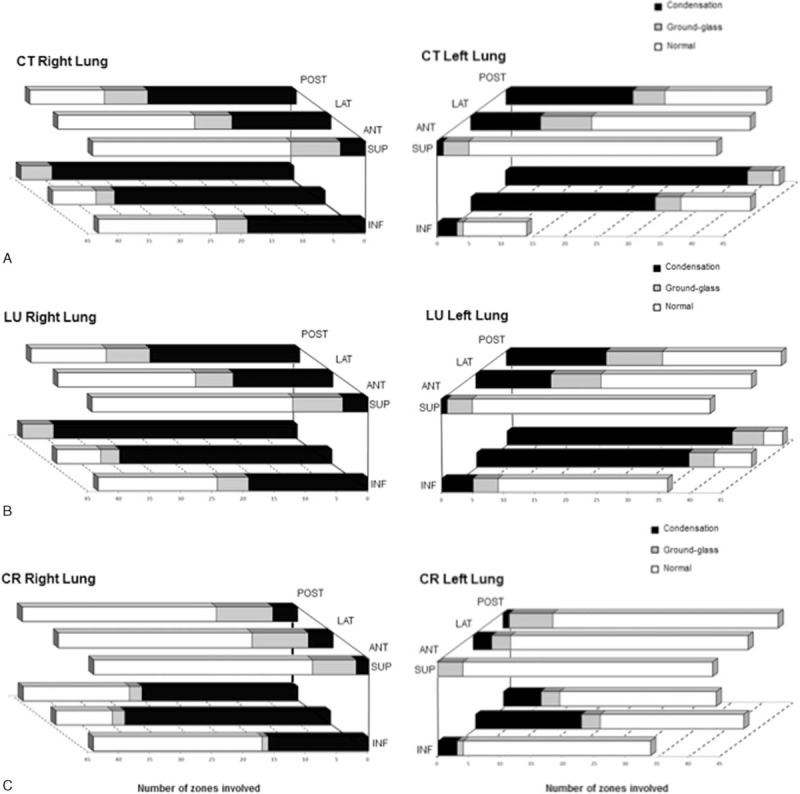
Numbers of zone involved of 3 patterns of lung parenchyma (consolidation, ground glass or B2 or alveolo-interstitial, and normal) according to each lung region during acute chest syndrome episodes. The anatomical distribution of lung opacities is presented in A, B, and C for CT, LU, and CR, respectively. Ant = anterior, CR = chest radiograph, CT = computed tomography, Inf = inferior, Lat = lateral, LU = lung ultrasound, Post = posterior, Sup = superior.

CT scans were performed within 1.0 (0.0–1.8) days of ACS onset. Bedside CR and LU were all performed within 24 h of CT scan. In the 44 ACS episodes included, a total number of 496 (94%), 507 (96%), and 519 (98%) lung regions could be assessed by CT scan, bedside CR, and bedside LU, respectively. The agreement between 2 readers of 115 LU recordings (in 10 randomly selected patients) was very high (0.85 ± 0.04). The anatomical distribution of lung opacities is shown in Figure 1A, B, and C for CT, LU, and CR, respectively. Consolidations were the most common pattern and prevailed in lung bases (especially postero-inferior regions). The agreement with CT scan lung patterns was significantly higher for LU as compared to CR (κ coefficients of 0.45 ± 0.03 vs 0.30 ± 0.03, *P* < 0.01 for the whole lung, and 0.47 ± 0.11 vs 0.06 ± 0.03, *P* < 0.01 in postero-inferior regions). The specificity for the detection of consolidated regions in the whole lung (using CT scan as the reference standard) was high for LU and CR (89% and 95%, respectively), whereas the sensitivity was high for LU but low for CR (72% and 44%, respectively); these findings were similar in postero-inferior regions: LU and CR had a specificity for the detection of consolidations of 82% and 91%, respectively, with a sensitivity of 94% and 45%, respectively.

Twenty-nine (66%) patients had a pleural effusion on CT scan, which involved the right pleura in 7 cases, the left pleura in 3 cases, and both sides in 19 cases. Using the CT scan as a reference standard, LU had a better accuracy for the diagnosis of pleural effusion as compared to CR, with a κ agreement coefficient of 0.73 ± 0.08 vs 0.06 ± 0.09, *P* < 0.001. The specificity for the detection of pleural effusion (using CT scan as the reference standard) was high for both LU and CR (81% and 73%, respectively), whereas the sensitivity was high for LU but low for CR (91% and 33%, respectively). Four pleural effusions were found large on LU (interpleural distance >25 mm).

### Loss of Aeration and Outcome

**FIGURE 2:**
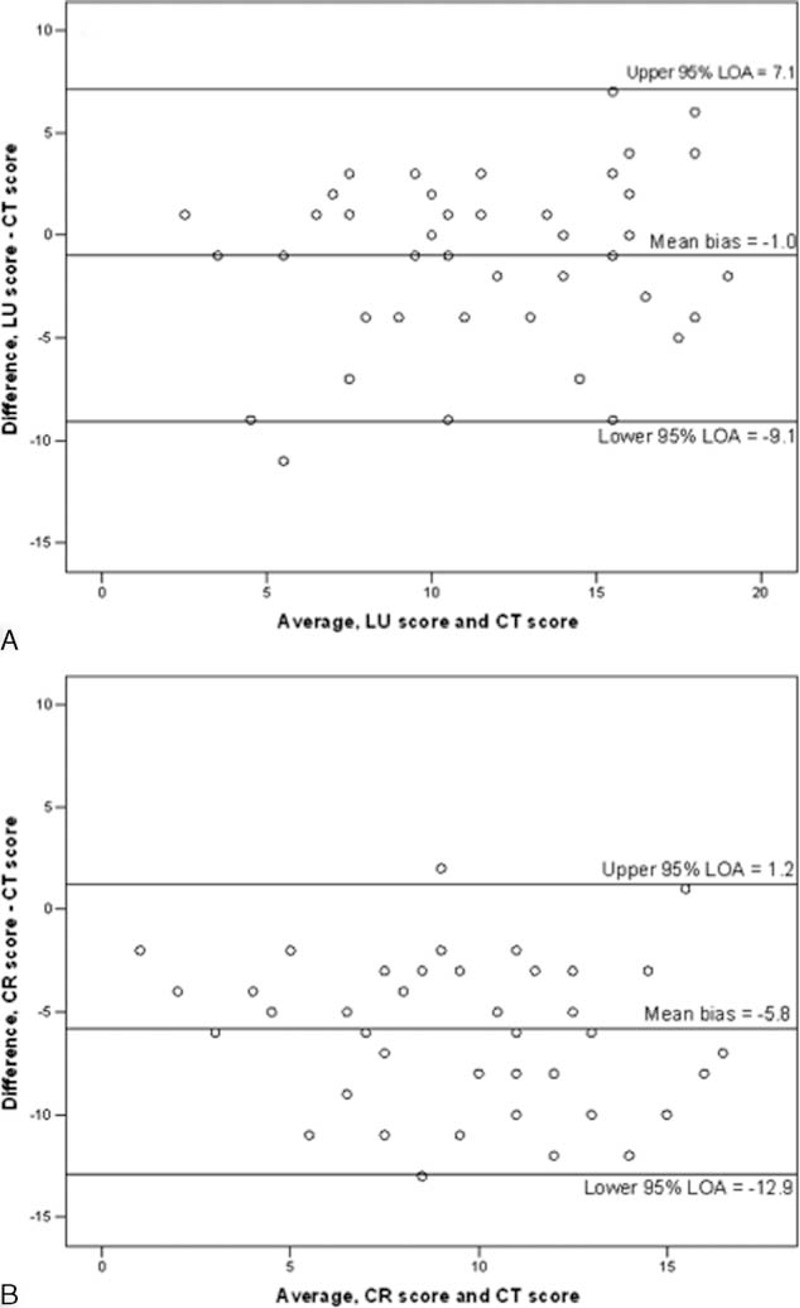
Bland–Altman plots of the agreement of computed tomography score with lung ultrasound score (A) and with chest radiograph score (B). In each plot, the horizontal lines represent (from above): upper 95% limit of agreement; mean bias; lower 95% limit of agreement. CR = chest radiograph, CT = computed tomography, LOA = limit of agreement, LU = lung ultrasound.

The median loss of aeration assessed by CT scan score, LU score, and CR score were 11.5 (10.0–15.0), 11.0 (8.0–15.0), and 6.0 (4.0–9.5), respectively. Loss of aeration assessed by CT scan score correlated significantly with that assessed with CR score (rho = 0.61, *P* < 0.001) or LU score (rho = 0.68, *P* < 0.001). The Bland and Altman analysis showed a nonfixed bias (*P* = 0.12) of −1.0 with 95% limits of agreement from −9.1 to 7.1 between LU score and CT score whereas CR score underestimated CT score with a fixed bias (*P* < 0.001) of −5.8 with 95% limits of agreement from −12.9 to 1.2 (Figure 2A and B). Patients with an LU score above the median value of 11 had similar baseline characteristics than those with lower LU scores except for a lower weight and body mass index (Table 1). Clinical and biological characteristics during ACS were similar between patients with higher values of LU scores and those with lower values, except for a higher respiratory rate at ACS diagnosis and higher peak platelet count during ICU stay in the former group (Table 2). There was no pleural effusion drainage. Treatments and outcomes of ACS are reported in Table 3 according to the extent of loss of lung aeration as assessed by the LU score. As compared to others, patients with a higher LU score had a larger volume of transfused and exsanguinated blood, greater oxygen requirements, more need for mechanical ventilation, and a longer ICU length of stay. In a sensitivity analysis restricted to the first ACS episode (n = 41), we found comparable results with a trend toward more need for mechanical ventilation (*P* = 0.06), and significantly higher values of transfused and exsanguinated blood, oxygen requirements, and ICU length of stay (*P* < 0.03 for all comparisons) in patients with a higher LU score as compared to others.

**TABLE 2:**
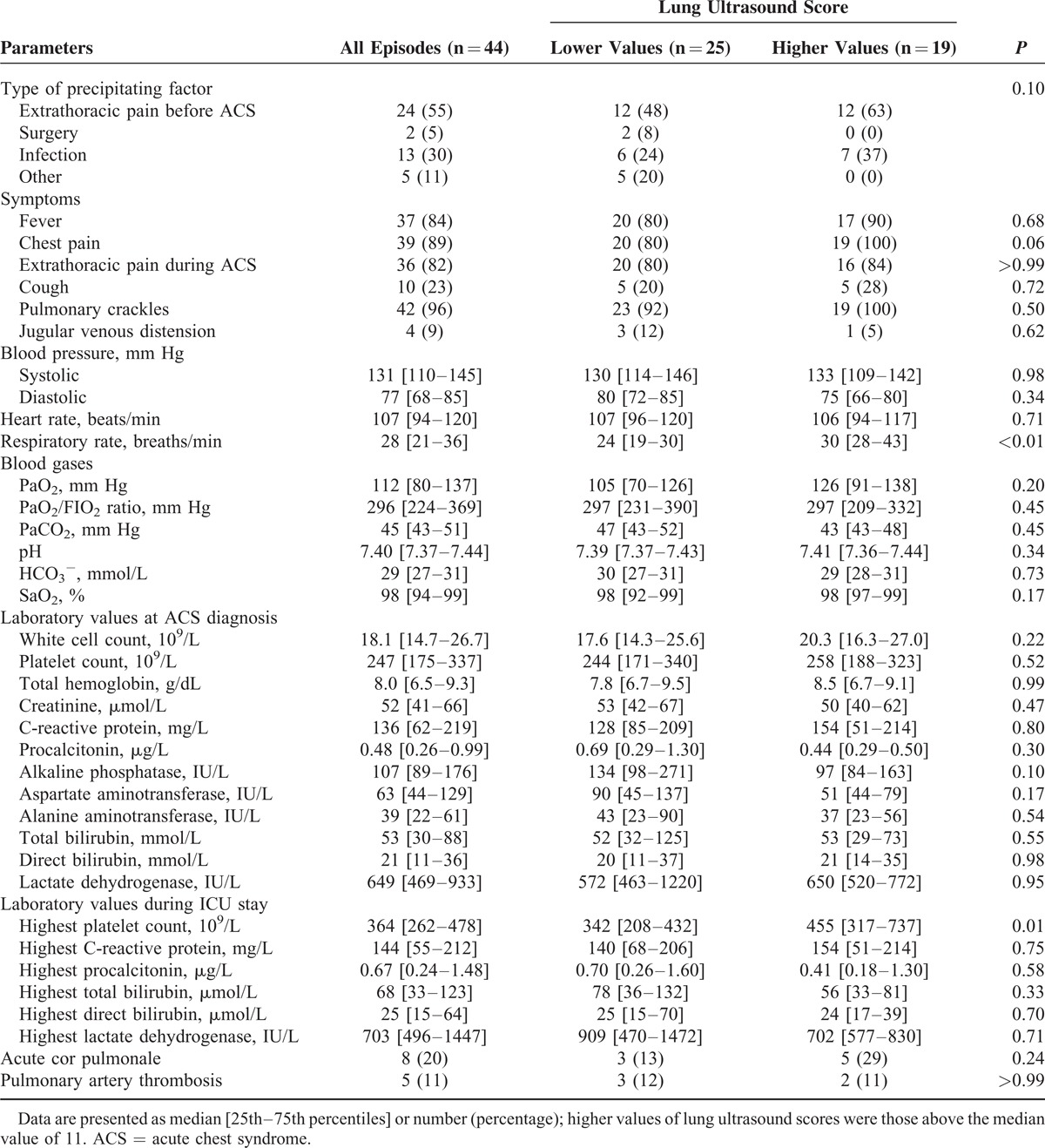
Clinical and Biological Data at Diagnosis and During Intensive Care Unit Stay in 44 Episodes of Acute Chest Syndrome, According to the Lung Ultrasound Score

**TABLE 3:**
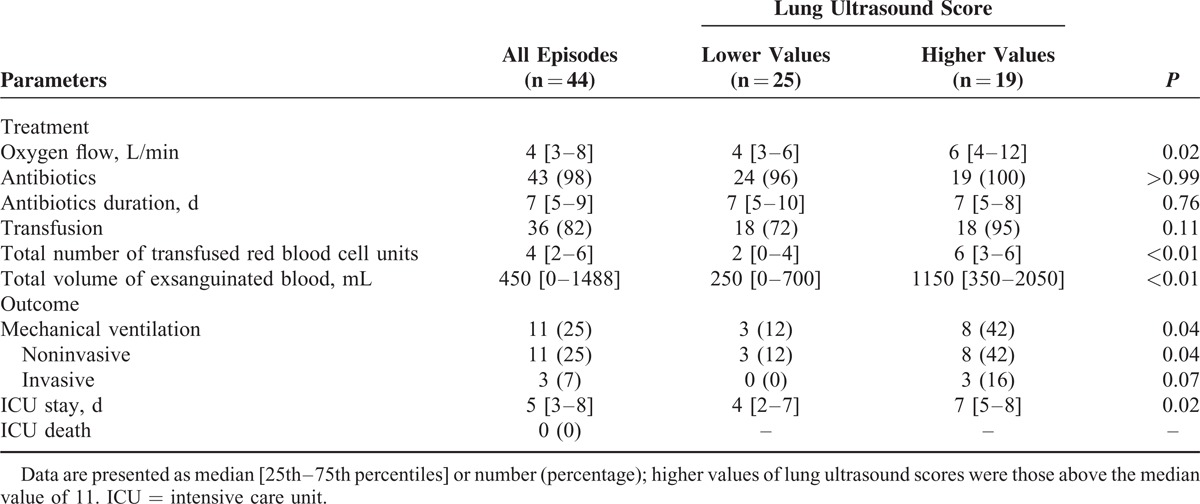
Treatment and Outcome of 44 Episodes of Acute Chest Syndrome, According to the Lung Ultrasound Score

## DISCUSSION

Our study is the first to assess bedside lung ultrasonography during ACS. LU had a good feasibility and reproducibility. It outperformed CR for the diagnosis of lung consolidation (especially in postero-inferior regions) and pleural effusion during ACS. The loss of lung aeration as assessed by the LU score was associated with a worse outcome.

The very good technical feasibility (98%) and reproducibility (85%) of LU found in our study is in line with previous reports in other settings.^20,21^ A recent study demonstrated that novice sonographers can perform LU and identify B lines adequately after a very short training course (30 min).^22^ As for other transthoracic ultrasound techniques, LU feasibility may be enhanced in thin patients as compared to obese^23^; this may explain the association between a higher LU score and a lower body mass index in our study. The better accuracy of LU as compared to CR for the detection of consolidated regions during ACS was driven by its superior sensitivity and was enhanced in postero-inferior regions. Our data confirm that lung consolidations are the most frequent opacities during ACS, with a clear basal preponderance in adults.^7,9,24^ In these regions, consolidations often reach the pleural surface, facilitating their detection by LU. LU also outperformed CR for the diagnosis of pleural effusion, as previously suggested in other settings.^12,25,26^ Radiological imaging has progressed toward an all-digital future, improving image presentation of bedside CR, but without major reduction in the radiation dose, as compared with screen film.^27^ The particularly poor sensitivity of CR for the detection of condensations (which were mostly basal) and pleural effusion probably highlights limitations of the silhouette sign.^28^ This drawback may also explain at least in part the systematic underestimation of the lung loss of aeration score by CR, using CT as the reference standard. Previous studies reported a similar advantage of LU over CR in patients with pneumonia,^29–31^ respiratory distress requiring mechanical ventilation,^32^ and those with the acute respiratory distress syndrome.^25,33^ The routine use of bedside LU in place of CR may allow reducing radiation exposure in sickle cell disease patients during their lifespan (inasmuch as ACS is typically a relapsing disease), while optimizing lung imaging capabilities. In centers implementing point-of-care ultrasonography, this technique may allow a rapid, readily available, and accurate tool for the follow-up of patients with ACS, as suggested for critically ill patients.^16^

A higher LU score was associated with a more severe lung involvement (as suggested by a higher respiratory rate) and a poorer outcome (more need for mechanical ventilation, larger volumes of transfusion and exsanguinated blood, and longer length of stay in ICU). In larger cohorts of adult patients with ACS, the need for mechanical ventilation predicted hospital deaths and resource utilization.^11,34,35^ Loss of lung aeration as assessed by the LU score may be useful for the early identification of patients at higher risk of poor outcome. Whether early implementation of a more aggressive treatment in such patients may alter ACS outcome needs further research. LU was shown to have a significant impact on decision making and therapeutic management in mechanically ventilated critically ill patients.^36^ The LU score could also be a pragmatic endpoint in studies assessing interventions aimed at altering ACS outcome, especially as the hospital mortality is relatively low.

Our study has some limitations. First, we included a relatively small number of selected patients; however, sickle cell disease is a rare disease and our results are robust in view of the 500 lung regions analyzed. We also excluded children and our findings may not be applicable to ACS during infancy, especially as the distribution of lung consolidations differs in adults and children with ACS, young children having more often isolated upper and middle lobe disease than adults and less often lower lobe disease.^37^ Future studies are needed for an external validation of our findings in a separate cohort. Second, using the silhouette sign to determine the posterior localization of the abnormalities is not optimal because opacities not in contact with hydric structures are impossible to localize; however, there is no available better alternative with antero-posterior bedside CR. Another technical limitation is the lack of Doppler study with LU; this modality may help assess shunt and its pharmacological modulation during ACS.^38^ Third, although all LU examinations were rapid and well tolerated in all patients (as previously reported in other settings), we did not precisely record duration nor quantified patient's tolerance. Last, the wide limits of agreement do not valid the interchangeability of CT and LU during ACS; however, the good feasibility, reproducibility, and clinical significance of LU allow its safe use in this setting.

## CONCLUSIONS

Our study is the first to assess LU diagnostic accuracy during ACS. LU outperformed CR for the diagnosis of lung consolidation and pleural effusion. The extent of loss of aeration, as assessed by the LU score, was associated with a worse outcome.
